# Supplementary material for: The Role of the Global Solar UV Index for Sun Protection of Children in German Kindergartens
Source: Children (Basel). 2022 Feb 3;9(2):198. doi: 10.3390/children9020198 (PMC8870228; doi:10.3390/children9020198)
Supplement: Supplementary file 1 [file children-09-00198-s001.zip › children-1557594-supplementary.pdf]

Supplemental Information

Table S1. Exposures perceived as risk factor of skin cancer among directors of kindergartens (N = 436). Exposures are grouped in true risk factors of skin cancer (upper part) and distracting factors (lower part). Absolute (n) and relative (%) frequencies of affirmative answers to the question whether the corresponding exposure acts as risk factor of skin cancer. Relative frequencies are accompanied by 95%-confidence intervals (95%-CI) calculated using Wilson's method.

Exposure	n	%[1]	95%-CI
Risk factors:			
Sunburns in childhood	252	57.8	53.1 – 62.4
Intermittent intensive sun exposure (e.g. during holidays in sunny areas)	251	57.6	52.9 – 62.1
Fair skin, fair hair	244	56.0	51.3 – 60.6
Chronic intensive sun exposure (e.g. during outdoor work)	241	55.3	50.6 – 59.9
Number of sunbaths during life	237	54.4	49.7 – 59.0
Number of nevi	235	53.9	49.2 – 58.5
Distracting factors:			
Rising air pollution	74	17.0	13.7 – 20.8
Nutrition	70	16.1	12.9 – 19.8
Allergies	58	13.3	10.4 – 16.8

[1] Denominator for all values was 436
